# Optimizing glycemic control in type 2 diabetes: the impact of the GLIDE program’s personalized digital health intervention

**DOI:** 10.3389/fcdhc.2024.1494009

**Published:** 2024-12-03

**Authors:** Anand Ambesange, Amit Lala Khomane, Jaymin Parikh, Devina Aswal, Mihir Gharia, Prachi Sharma, Vishva Trivedi, Twinkle Maheshwari, Astha Mishra, Bhavan Bhavsar, Vrushali Athavale

**Affiliations:** ^1^ Internal Medicine, Anand Nursing Home, Mumbai, India; ^2^ Internal Medicine, The Clinic, GM House Thane, Thane, India; ^3^ Medical Affairs, Digicare Health Solutions Private Limited, Ahmedabad, India

**Keywords:** digital health program, glycemic control, personalized intervention, root cause analysis, type 2 diabetes mellitus

## Abstract

**Background:**

The integration of digital health applications into type 2 diabetes mellitus (T2DM) management presents promising opportunities for optimizing glycemic control, enhancing adherence, and improving health outcomes. MyTatva’s Glycemic Lifestyle Intervention in Diabetes Empowerment (GLIDE) program, which integrates dietary and exercise regimens, cognitive behavioral therapy (CBT), and Internet of Things (IoT) devices, potentiates this approach. This study aimed to evaluate the effectiveness of the GLIDE program’s personalized, comprehensive approach in improving glycemic control over 90 days among T2DM patients.

**Methods:**

During the study period, 30 diabetic patients completed their GLIDE journey with expert dieticians, physiotherapists, and behavior therapists. Each patient received a personalized root cause analysis based on lifestyle assessment and disease-specific parameters. Statistical analysis was conducted using a paired t-test on the deidentified HbA1c, FBS, and PPBS data at baseline and post-intervention.

**Results:**

Throughout the study, 27 patients actively adhered to the GLIDE program. All the parameters showed statistically significant (p<0.05) changes post-intervention. HbA1c decreased by 11.79% from 8.43% ± 1.32 to 7.44% ± 0.64. Significant reductions were observed in PPBS (47.7%), decreasing from 260.89 mg/dL ± 36.31 to 136.27 mg/dL± 6.36, compared to FBS (31.1%), which decreased from 8.43 mg/dL± 1.32 to 7.44 mg/dL± 0.64.

**Conclusions:**

The effectiveness of the GLIDE program is based on a comprehensive root cause analysis approach. The detailed analysis of the patient’s clinical journey by health experts at regular intervals enables precise goal management, resulting in expected outcomes for better glycemic control. Therefore, personalized digital health plans are vital for achieving clinically significant changes.

## 1 Introduction

Type 2 diabetes mellitus (T2DM) is characterized by persistent elevated blood glucose levels, with clinical guidelines emphasizing for glycated hemoglobin (HbA1c) levels to be at 7.0% or lower for most diabetes patients. Approximately half of T2DM diagnosed patients fail to maintain this target facing the increased risk of developing macrovascular and microvascular complications (1, 2). These data highlight that medication alone may not be an effective long-term strategy for disease management. The successful control of T2DM requires a healthy lifestyle, where behavioral modification play a crucial role in managing T2DM and controlling disease progression (3, 4). Despite the critical importance of a lifestyle modification, many patients continue to struggle with implementing these essential behavioral modifications effectively due to lack of education, insufficient support, and inadequate personalized feedback (5).

Traditional methods to bring out behavioral modifications include educational sessions, behavior change techniques, group exercises, and therapy sessions. However, these methods are limited to face-to-face interactions, lack of real-time data, and overlook individual needs (6). Digital health programs (DHPs) represent an emerging technology in transforming patient care by leveraging digital platforms to help deliver remote lifestyle modification support to patients (7, 8). DHPs offer healthcare providers a platform to tailor interventions to the unique needs and circumstances of individuals, taking into account factors such as comorbidities and socio-economic backgrounds. This personalized approach enhances patient engagement and fosters better adherence to treatment plans (9). Continuous monitoring through IoT (Internet of Things) devices, which are interconnected digital devices that collect and exchange data over the internet, enables real-time monitoring, alerts for abnormal readings, and suggestions for treatment adjustments (10). Teleconsultations with healthcare providers are also facilitated, enabling remote guidance and support. However, it is not without its own set of challenges; consistent engagement and adaptation among the users are critical for the success of digital health programs.

Considering these challenges, The Glycemic Lifestyle Intervention in Diabetes Empowerment (GLIDE) program is a comprehensive, digital health program for T2DM management through targeted lifestyle modifications. The GLIDE program integrates a multidisciplinary team, including a physiotherapist, dietitian, and cognitive behavioral therapy (CBT) coach, to deliver personalized intervention plans tailored to individual needs. The program emphasizes continuous user engagement and adaptability, supported by real-time monitoring through the use of a Body Composition Analysis (BCA) machine for tracking progress. In this study, we aim to evaluate the efficacy of the GLIDE program in improving glycemic control and lifestyle changes in T2DM patients.

## 2 Methods

### 2.1 Research objective

This study aimed to evaluate the effectiveness of MyTatva’s GLIDE program in improving glycemic control among patients diagnosed with T2DM over a 90-day period. The primary objective includes improvement in key glycemic parameters, including mean HbA1c, fasting blood sugar (FBS), and postprandial blood sugar (PPBS) levels. The secondary objective is to evaluate changes in weight, Body Mass Index (BMI), waist circumference, and body fat percentage.

### 2.2 Study design and participants

This is a retrospective, preliminary, real-world evidence study based on the analysis of deidentified patient data. Patients onboarded onto MyTatva’s GLIDE program included patients, either through direct referrals by their treating physicians or via social media platforms. The inclusion criteria comprised individuals with a diagnosis of T2DM with HbA1c levels >6.5%, aged ≥18 years, owning a smartphone with internet access, possessing a minimum level of literacy in English to comprehend the program, and were able to read and provide informed consent to participate. Patients with active or recent (<90 days) participation in lifestyle intervention programs or use of weight-loss supplements, recent surgical procedures, pregnancy or breastfeeding within the past 3 months, and a history of unstable angina pectoris or stroke within the last 6 months were excluded from the study.

### 2.3 Ethical considerations

The study received approval from the ACEAS-Independent Ethics Committee with Protocol number MTD120324. The study was conducted in accordance with the Declaration of Helsinki and its subsequent revisions.

### 2.4 GLIDE program

The GLIDE program is a multi-phased, patient-centric approach for managing T2DM and adapting to healthy lifestyles. Once patients are onboarded on the MyTatva application, they complete a comprehensive questionnaire aimed at evaluating diverse clinical parameters, dietary habits, status of physical activity, sleep patterns, stress levels, smoking, and alcohol consumption. Health coaches, including physiotherapists, CBT coach, and dieticians, then conduct a root cause analysis focusing on aspects of the patient’s lifestyle that are compatible with effective T2DM management. Based on the comprehensive assessment, a personalized diet chart was formulated, focusing on gradual and sustainable changes to the participant’s eating habits. The initial dietary modifications were designed to be subtle, targeting specific behaviors that could lead to healthier eating habits. A personalized exercise plan was developed to complement with personalized dietary chart. The exercise plan considered participants’ baseline physical activity levels and existing physical limitations, with recommendations considering their lifestyle and job requirements. For those with sedentary jobs, the plan included strategies like walking breaks, stretching exercises, and desk workouts. For participants with jobs involving regular travel, the plan provided adaptable exercise routines suitable for various settings. The exercise plan focused on gradually increasing the intensity and duration of physical activity to build the participant’s endurance and strength. As participants progressed, the plan was adjusted to include more vigorous activities considering their adherence and endurance to current plan. The CBT component of the program was designed to support participants in developing a positive mind set and addressing psychological barriers to lifestyle changes. The CBT coach provided regular follow-up sessions to monitor the participant’s progress and offer continuous support. Through these sessions, participants were taught various coping strategies to manage stress and emotional eating. Techniques such as mindfulness, relaxation exercises, and cognitive restructuring were employed to challenge and change negative thought patterns. Enhancing motivation was another key aspect of the CBT sessions, where participants were encouraged to stay committed to their diet and exercise plans by setting realistic goals and celebrating small achievements. For participants who struggled with initial changes in their diet and exercise patterns, the CBT coach provided additional support and tailored interventions. The focus was on behavioral change, ensuring that participants could transition smoothly and effectively towards healthier lifestyle choices.

**Figure 1:**
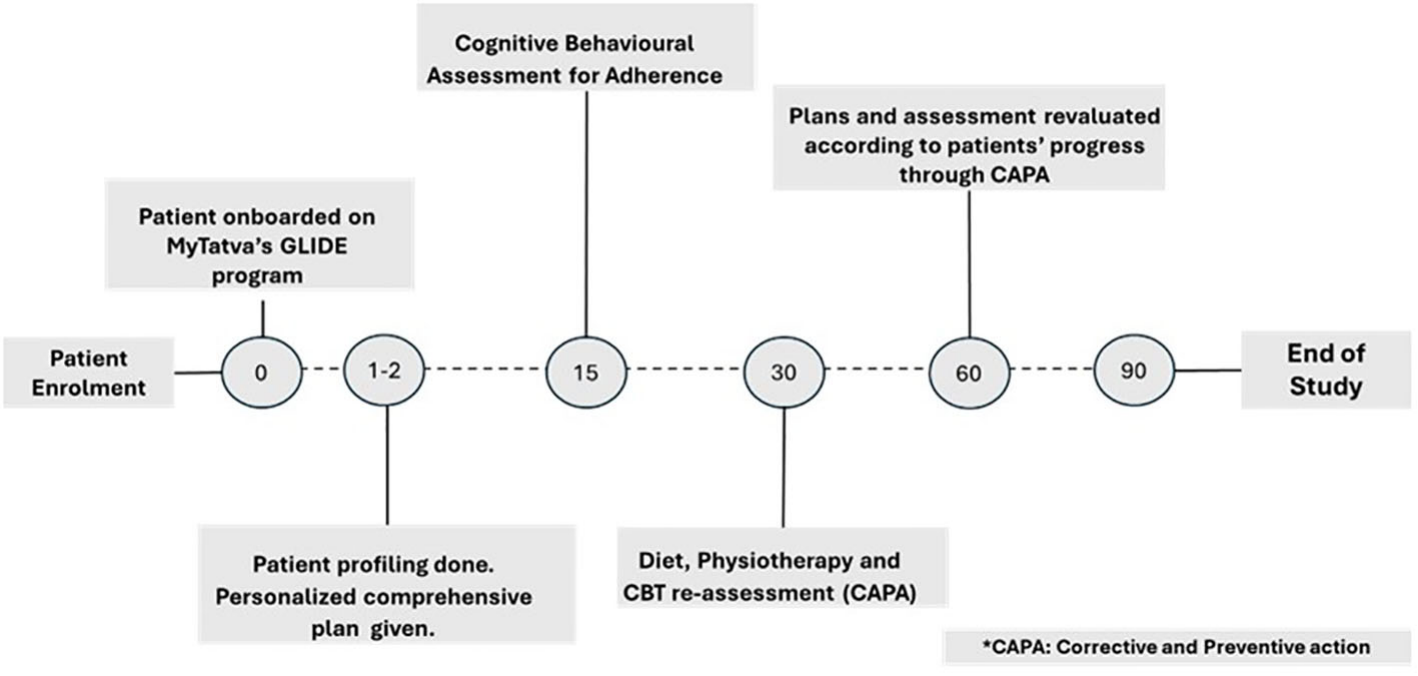
Overview of patient’s GLIDE program 90 days study.

At 15-day intervals, the CBT coach assesses the patient’s adherence to the exercise and diet plan, making necessary revisions based on data captured through IoT devices on the MyTatva app. After 1 month, patients with 80% or higher adherence receive an advanced plan, while those with lower adherence are evaluated for obstacles and given tailored support. Regular follow-ups from all coaches ensure continuous progress tracking, plan adjustments, and addressing patient concerns. This dynamic process, driven by remote monitoring and patient feedback, leads to measurable improvements in glycemic and BCA parameters after the 90-day program (**Figure 1**).

### 2.5 Data collection and statistical analysis

To facilitate comprehensive data collection, patients were provided with a Body Composition Analyzer (BCA) machine and digital food diary integrated into MyTatva app. This device enabled auto-fetching of patients BCA measurements regularly, ensuring continuous real-time monitoring of their data. The data collected was organized in a Microsoft Excel spreadsheet. Analysis was conducted using Ver. 25.0 of IBM SPSS (Statistical Package for the Social Sciences). Quantitative data were expressed as means and standard deviations, and significance were established at a p-value <0.05, adhering to a 5% significance level.

## 3 Results

### 3.1 Patient demographic and baseline parameters

**Figure 2:**
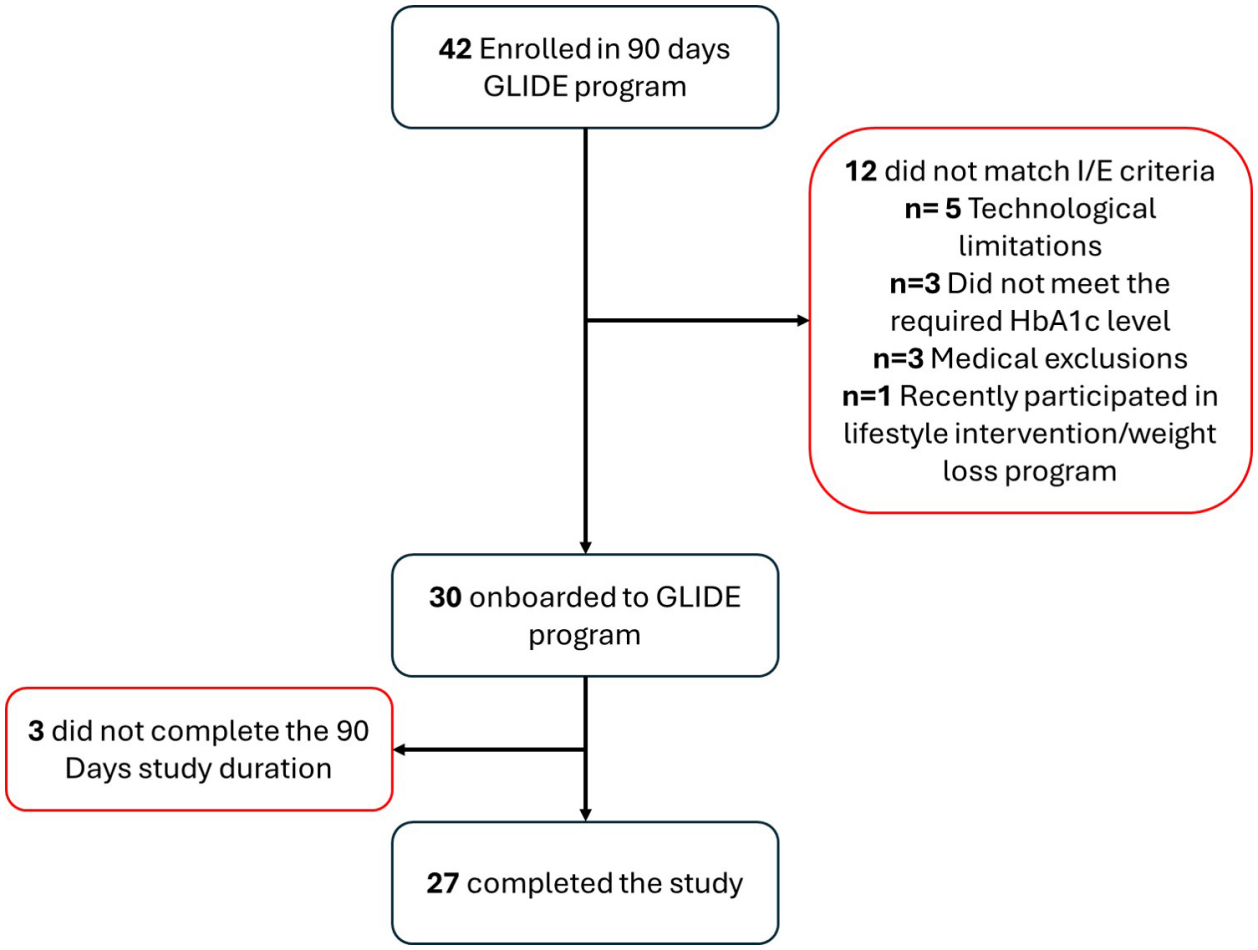
Patients flow.

Demographic characteristics revealed a female predominance of 56% (n=15) with mean age of 57 + 9, and the mean HbA1c at baseline was 8.43 ± 1.32%, for most patients with T2DM. The cohort was predominantly employed, all residing within metropolitan area. Other demographic and baseline parameters are summarized in **Table 1**. During the study duration between December 2023 to January 2024, 42 patients were subscribed to or recommended to the app, out of which 27 actively adhered to 90 days study duration (**Figure 2**).

**Table 1:** Baseline characteristics of the study participants (n=27).

Parameters	Baseline (n=27)
Female, n (%)/male, n (%)	15 (56%)/12 (44%)
Age years (mean ± SD)	57± 9
Comorbidities present (n%)	15 (55%)
Years since diagnosis (Mean ± SD)	11 ± 8
Region and residence	
North India, n (%)	5 (18.5%)
South India, n (%)	11 (40.8%)
West India, n (%)	6 (22.2%)
East India, n (%)	5 (18.5%)
Within Metropolitan Area	27 (100%)
Employment status	
Student, n (%)	0 (0%)
Housewife, n (%)	4 (14.9%)
Employed, n (%)	17 (62.9%)
Self-employed, n (%)	3 (11.1%)
Retired, n (%)	3 (11.1%%)
Clinical parameters	
%HbA1c (mean ± SD)	8.43 ± 1.32
FBS mg/dL (mean ± SD)	175.08 ± 26.55
PPBS mg/dL (mean ± SD)	260.89 ± 36.31
BCA parameters	
Weight (kg) (mean ± SD)	80.94 ± 9.48
Waist circumference (cm) (mean ± SD)	53.5 ± 25.11
BMI (kg/m^2^) (mean ± SD)	25.52 ± 7.57
Body fat % (mean ± SD)	23.57 ± 5.2
Total cholesterol, mg/dL (mean ± SD)	178.18 ± 32.75
Triglycerides, mg/dL (mean ± SD)	110 ± 26.2

### 3.2 Change in glycemic parameters

Following a 90-day program GLIDE program, a significant mean absolute reduction in HbA1c levels by 0.99% (p<0.05) was observed (**Table 2**). After the end of the program, seven participants observed a reduction in HbA1c level of more than 1%, with four patients achieving HbA1c level of below 7%. In this study, seven patients had HbA1c levels of ≥8, and an average of 2.6% reduction in HbA1c level was observed among them. A statistically significance decrease in mean FBS level by 54.46 mg/dL (p<0.05) and PPBS level by 124.62 mg/dL (p<0.05) was observed (**Table 2**).

**Table 2:** Changes in glycemic parameters.

Parameter	Baseline	End of study (90 days)	Mean difference	Percentage change	p-Value
**HbA1c**	8.43 ± 1.32	7.44 ± 0.64	0.99	11.79%	<0.05
**FBS**	175.08 ± 26.55	120.62 ± 22.05	54.46	31.11%	<0.05
**PPBS**	260.89 ± 36.31	136.27 ± 6.36	124.62	47.77%	<0.05

### 3.3 Change in BCA parameters

Participants showed a mean weight reduction of 2.84 kg after completion of the program. Weight reduction was observed in 51% of patients with 29% reporting weight loss of >1 kg. This decrease in weight was accompanied by a mean reduction of 1.21 kg/m^2^ in BMI (**Table 3**). A statistically significant reduction was observed in the frequency of eating out or ordering takeout meals, decreasing from 5.58 ± 6.77 at baseline to 3.02 ± 4.44 at the end of the study (p = 0.01).

**Table 3:** Change in BCA parameters.

Parameters	Baseline	End of study (90 days)	Mean change
**Weight (kg) (mean ± SD)**	80.94 ± 9.48	78.1 ± 9.67	−2.84
**Waist circumference (cm) (mean ± SD)**	53.5 ± 25.11	52.1 ± 25.11	−1.4
**BMI (kg/m^2^) (mean ± SD)**	25.52 ± 7.57	24.31 ± 1.06	−1.21
**Body fat % (mean ± SD)**	23.57 ± 5.2	22.2 ± 5.55	−1.37
**Total cholesterol, mg/dL (mean ± SD)**	178.18 ± 32.75	176.11 ± 23.1	−2.07
**Triglycerides, mg/dL (mean ± SD)**	110 ± 26.2	108.7 ± 24.56	−1.3

**Figure 3:**
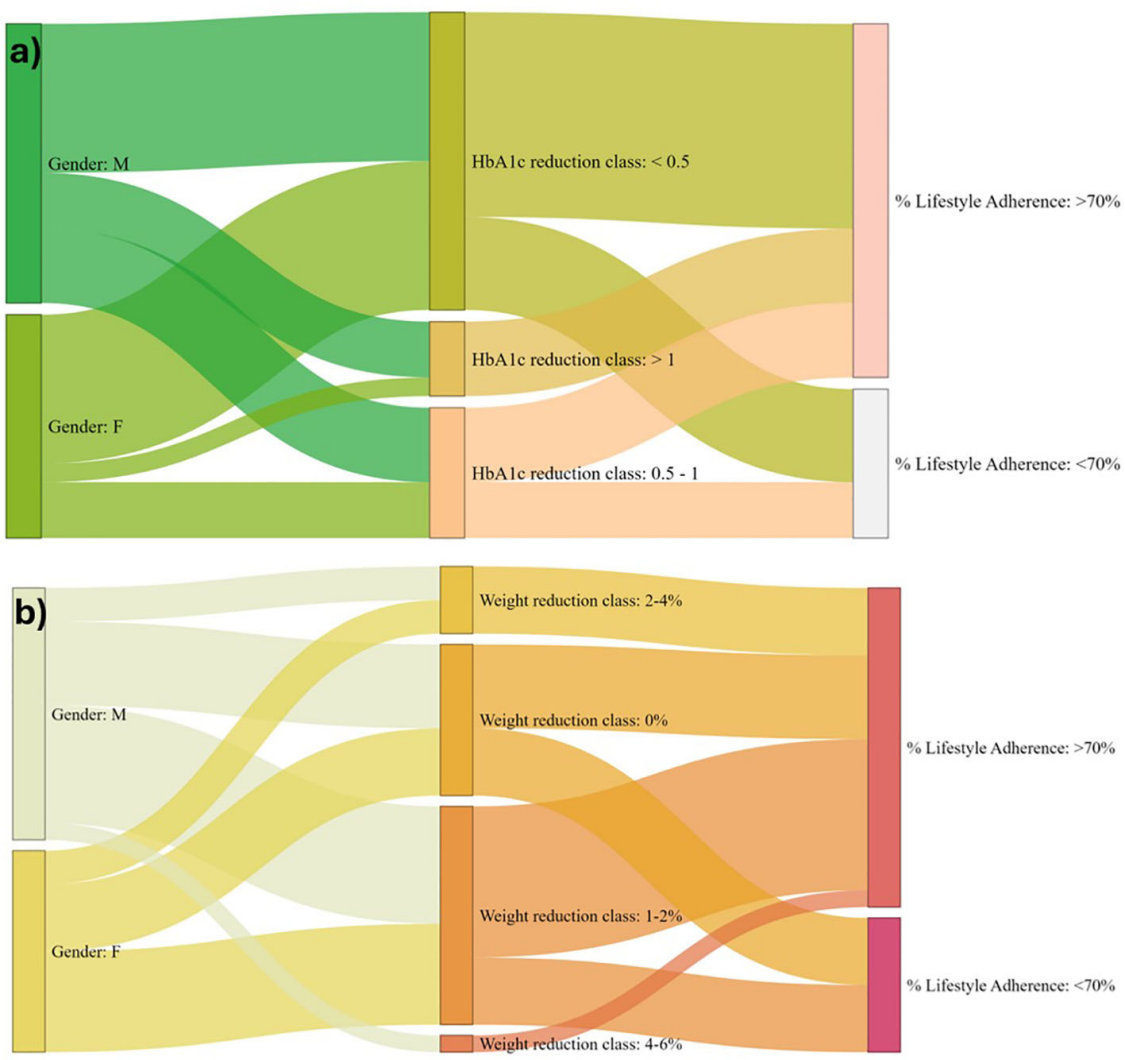
**(A)** Sankey’s diagram showing the correlation between the reduction in HbA1c level in respect to their lifestyle adherence; **(B)** Sankey’s diagram showing correlation between weight reduction in respect to their lifestyle adherence.

**Figure 3A** shows the relationship between gender, HbA1c reduction, and lifestyle adherence. It illustrates the flow from gender to three categories of HbA1c reduction: <0.5%, between 0.5% and 1%, and >1%. The final step displays how these groups align with lifestyle adherence, classified as either ≥70%. This flow helps visualize how different HbA1c reductions correspond to varying adherence levels in patients. **Figure 3B** shows the flow from gender to different categories of weight reduction. Patients are categorized into four groups based on their weight reduction: 0%, 1%–2%, 2%-4%, and 4%–6%. The chart then connects these weight reduction categories to lifestyle adherence, which is classified as ≥70%. This flow helps visualize how weight loss correlates with adherence to lifestyle changes in patients.

### 3.4 Patient feedback surveys

To further optimize the feedback collection process, the GLIDE program incorporates end-user feedback to gather valuable insights, enhancing user experience and satisfaction levels. The participation of 27 patients in surveys yielded an overall Customer Satisfaction Score (CSAT) score of 93%, indicating a high level of satisfaction.

### 3.5 Program engagement and glycemic control

Participants logged into the app an average of 4.3 times per day and spent an average of 81.76 min over 90 days. A strong correlation was found between patient engagement and HbA1c reduction. The number of app visits per day showed a correlation coefficient of 0.8201 (p<0.0001; 95% CI, 0.63–0.92). Similarly, the time patients spent on the app per day demonstrated a correlation coefficient of 0.8305 (p<0.0001; 95% CI, 0.65–0.93).

## 4 Discussion

The findings from this study show the effectiveness of MyTatva’ GLIDE program in improving glycemic control and promoting a healthy lifestyle among patients with T2DM. Over 90 days intervention, patients showed significant improvement in key glycemic parameters with absolute reduction of 0.99% in HbA1c levels. A significant correlation was observed between participant engagement in the program and HbA1c level reduction after the program. Clinical studies have shown that a 1% reduction in mean HbA1c levels is associated with a reduction in the risk of microvascular complications by 37%, death related to diabetes by 21%, and myocardial infarction by 14% (11).

The GLIDE program’s key feature is the root cause analysis of each patient that is hindering them from treatment adherence and healthy lifestyle maintenance along with real-time monitoring, which allows patients, physicians, and health coaches to be aware of changes in glucose levels. Dietary intake is the major determinant of blood glucose levels, and each patient has a different glycemic response even after eating identical meals (12). Thus, it is important for patients to be aware of their meal planning and effect that it may have on their glycemic response. To address these challenges, GLIDE offers tailored interventions by dieticians and physiotherapists to develop personalized dietary plan and exercise program by evaluating the baseline health score from detailed questionnaires filled by patients. The evidence strongly supports the importance of physical exercise and healthy diet in managing HbA1c levels in individuals with notable reductions of 0.2%–1.0% (13–16). In addition, GLIDE program also provides CBT interventions, which ensures the consistency of adherence to personalized goal. Studies have demonstrated that CBT interventions have led to improved glycemic control, self-care behavior, and general health among individuals with T2DM. As per a study reported by Pan et al. (2018), a reduction in HbA1c levels by 0.6% was observed (17), while a study by T. Alanzi et al. (2022) found a decrease in HbA1c levels by 0.38% (18). CBT has also proved to be effective in implementing lifestyle changes especially for weight loss (19). In the context of Indian digital health program studies, limited data exist where CBT intervention and its impact of glycemic control is discussed, as behavioral changes play important role in patient’s adherence to treatment plan. The app’s library of regular educational blogs and videos likely played a key role in maintaining high levels of engagement, which was shown to have a strong association with HbA1c reduction. High engagement was correlated with greater reductions in HbA1c, demonstrating the importance of patient involvement in managing their condition.

Management of T2DM through digital health intervention is an ongoing effort, and the outcomes reveal valuable insight into how different approaches are affecting patients. In a study reported by Zimmerman et al. (2021), improvement in HbA1c level of 1.1% was observed, and their program incorporated health coach, dietitian, or diabetes care and education specialist, with 27.2% of patients adhering to the treatment plan (20). In contrast, a study of Joshi et al. (2023) reported higher improvement of 1.2% in HbA1c level; their intervention included nutritionist, physiotherapist, and psychotherapist, with 30% patient’s adherence (21). In contrast, the study reported by Berman et al. (2018) (22) showed 0.9% of HbA1c reduction with intervention of change in dietary habits and effect of plant-based diet with patients’ adherence of 92%. The study by Hsia et al. (2022) (23), which included the intervention of CBT coach only reported the HbA1c reduction of 0.28% with 85% of patients adhering to the treatment plan. In respect to that, the present study program incorporated the dietician, physiotherapist, along with CBT. In a comprehensive attempt, introducing CBT coach improved patient’s adherence to treatment plan with 92% of patients showing improvement in HbA1c level in contrast to other studies where 85.3% (22) and 57% (23) patients showed improved HbA1c levels.

The integration of incident reporting within the GLIDE program empowers patients to promptly report adverse events, ensuring safety and efficacy in interventions. The patients in this study duration reported no incidents from the app incident reporting system, and the overall CSAT score of 93% from participants indicates high satisfaction with the GLIDE program.

The GLIDE program’s personalized and adaptive approach has been key to sustaining patient engagement and driving improvements in lifestyle and clinical outcomes. By offering individualized diet, exercise, and cognitive behavioral therapy plans, the program addresses both the physical and psychological needs of patients. Continuous monitoring through IoT devices and regular follow-ups ensure that care plans are dynamically adjusted, fostering accountability and keeping patients engaged. The reduction in eating out or ordering takeout coupled with increased uptake of nutritionally rich food with reflects the program’s success in promoting healthier dietary habits. These changes, coupled with tailored physical activity plans, contributed to improvements in BCA parameters. Additionally, adherence to the CBT component played a significant role in sustaining patient motivation and commitment. This comprehensive approach led to a notable improvement in glycemic control. While the GLIDE program shows promise, several limitations should be noted. The lack of a control group makes it difficult to attribute the improvements solely to the program. The small sample size (n=30) reduces the generalizability of the findings, and the short duration of 90 days does not capture the long-term sustainability of the improvements. Additionally, the requirement for smartphone access and English literacy may introduce selection bias, limiting the program’s applicability to broader populations. Future studies should include larger samples, control groups, and extended follow-up to better assess the program’s long-term effectiveness.

## 5 Conclusion

The GLIDE program demonstrated significant improvements in glycemic control and lifestyle changes among T2DM patients through personalized interventions integrating diet, exercise, and cognitive behavioral therapy (CBT). With a 0.99% reduction in HbA1c and substantial reductions in FBS and PPBS levels, the program’s comprehensive, real-time monitoring and tailored support ensured high patient engagement and adherence. While the study’s small sample size, short duration, and lack of a control group present some limitations, the findings highlight the potential of digital health programs to effectively manage T2DM, warranting further research in larger and more diverse populations.

## Data Availability

The original contributions presented in the study are included in the article/supplementary material. Further inquiries can be directed to the corresponding author.
